# Progress in bioleaching: part B, applications of microbial processes by the minerals industries

**DOI:** 10.1007/s00253-022-12085-9

**Published:** 2022-08-30

**Authors:** Francisco F. Roberto, Axel Schippers

**Affiliations:** 1 Technical Services Processing and Metallurgy, Newmont Corporation, Englewood, CO USA; 2 Federal Institute for Geosciences and Natural Resources (BGR), Geomicrobiology Unit, Resource Geochemistry, Stilleweg 2 30655, Hannover, Germany

**Keywords:** Bioleaching, Biooxidation, Biomining, Copper, Nickel, Cobalt, Reductive bioleaching, In situ leaching, Laterite

## Abstract

**Abstract:**

This review provides an update to the last mini-review with the same title pertaining to recent developments in bioleaching and biooxidation published in 2013 (Brierley and Brierley). In the intervening almost 10 years, microbial processes for sulfide minerals have seen increased acceptance and ongoing but also declining commercial application in copper, gold, nickel and cobalt production. These processes have been applied to heap and tank leaching, nowadays termed biomining, but increasing concerns about the social acceptance of mining has also seen the re-emergence of in situ leaching and quest for broader applicability beyond uranium and copper. Besides metal sulfide oxidation, mineral dissolution via reductive microbial activities has seen experimental application to laterite minerals. And as resources decline or costs for their exploitation rise, mine waste rock and tailings have become more attractive to consider as easily accessible resources. As an advantage, they have already been removed from the ground and in some cases contain ore grades exceeding that of those currently being mined. These factors promote concepts of circular economy and efficient use and valorization of waste materials.

**Key points:**

*• Bioleaching of copper sulfide ore deposits is producing less copper today*

*• Biooxidation of refractory gold ores is producing more gold than in the past*

*• Available data suggest bioleaching and biooxidation processes reduce carbon emissions*

## Introduction

The first report in this recurring series on microbial processes in the minerals industries was published in 2003 (Olson et al. 2003), followed by an update after 10 years (Brierley and Brierley 2013) with bioleaching and biooxidation of sulfide minerals, primarily copper (in the case of bioleaching) and refractory gold ores (biooxidation) matured to represent significant production of those metals in the mining industry. Some extension of bioleaching had been made to other base metals (cobalt, nickel) and stirred tank operations used for biooxidation had also been extended to bioleaching of base metal concentrates. The mining industry’s understanding of the practical considerations of deploying microbial sulfide oxidation flowsheets had become generalized (The Chemistry of Gold Extraction 2nd Edition, 2006; SME Mineral Processing and Extractive Metallurgy Handbook, 2019) although the rigor of maintaining viable microbial processes and the microorganisms driving those processes might be underappreciated. Nearly a decade has elapsed since the last review update in 2013, and the realities of depleting mineral resources and deepening mines now mean that the ideal ore bodies on which to practice these microbial processes are disappearing, and other alternative, non-microbiologically based flowsheets are beginning to supersede bioleaching to economically exploit the available resources, especially primary copper sulfides. While the mini-review part A focusses on microbiology and bioleaching mechanisms (Vera et al. 2022), this mini-review part B provides an overview on biomining including metal production data. Particular biohydrometallurgical processes and related microbiological background are thoroughly described in a recently published book (Johnson et al. 2022).

The first section of this mini-review will update the status of industrial projects that are either new or for which some details were provided by Brierley and Brierley in 2013, where those projects continue. Projects known to no longer be using or considering a bioleach or biooxidation strategy have largely been eliminated but, in some cases where new strategies are being deployed, remain part of the list. The second section of the mini-review will focus on new applications of microbial processes to mineral extraction, including (a) in situ recovery, (b) reductive bioleaching under acidic conditions, and (c) valorization of mine wastes (tailings and acid rock drainage) and industrial wastes.

## Copper bioleaching

At the time that this 2022 article is being concluded, the spot price of copper had achieved an all-time high of over US $11,067/tonne ($5.02/lb). This is an incredible rebound after a long decline to a low of US $4863/tonne in 2016, and a similar recent low in March of 2020 resulting from the COVID-19 pandemic and global economic slowdown. The previous all-time high of US $10,512/tonne ($4.90/lb) was achieved on May 2021 which indicates that copper price remains extremely volatile and sensitive to global demand and events that influence that demand.

Since the last published version of this applied report, Chile has emerged as the undisputed global leader in copper production, followed by Peru, China, Democratic Republic of Congo, and the USA. All five leading countries exceed 1 million tonnes of annual production, but Chile leads by far with 2020 production approaching 6 million tonnes, nearly one-third of total global production. These rankings may change with new operations expanding the production of copper in Australia and other parts of Africa.

Besides being the largest contributor to global production, the Chilean copper industry can be considered to reflect the overall situation of the copper industry today, both from the perspective of diminishing, easy to extract resources as well as a shift to increasingly larger operations and changing process flowsheets to accommodate changing mineralogy. Much of the copper production capacity that has been developed in Chile was well-suited to a large copper oxide resource that could be readily extracted with sulfuric acid, with the resulting loaded solution as an ideal feed solution for solvent extraction-electrowinning (SX-EW) plants producing high-purity copper cathodes. This process strategy also lent itself to effectively handling the process effluents of a bioleach process to extract copper from secondary copper sulfides like covellite and chalcocite. For that reason, Chile was able to broadly apply bioleaching at several of the largest copper mines in that country, including Cerro Colorado, Chuiquicamata, Collahausi, Escondida Norte, Quebrada Blanca, Spence, and Zaldivar, to name many of the largest operations (Table 1). It was estimated that as much as 42% of Chilean copper production from SX-EW in 2010 was attributable to bioleaching (Schippers et al. 2014). Copper cathode produced from SX-EW represented 38.5% of annual production in Chile that year (https://www.cochilco.cl/Paginas/English/Statistics/Data%20Base/Mining-Production.aspx; copper production by product type), meaning about 16% of total Chilean copper production via bioleaching for the year 2010. It can also be seen in Table 1 and previously published works that the operators of these mines included several of the world’s largest copper producers, including Codelco, the national copper company of Chile, Teck, Glencore, Anglo American, BHP, Rio Tinto, Vale, Barrick, Grupo Mexico, and Freeport McMoRan (Schippers et al. 2014).

**Table 1:** Current copper bioleach operations including several that are presumed no longer to utilize bioleaching or are not strictly bioleaching operations (production listed in parentheses)

Mine	Location	Operator	Cathode Cu production, t/y	Year Initiated	Notes
Carmen de Andacollo	Chile	Teck/ENAMI	1,000	1996	Declined from a historic production level of 58,000 t/y
Doña Inés de Collahuasi	Chile	Anglo American/Glencore/Mitsui	56,619	1995	Cathode copper production represents 9% of 2020 mine production
Escondida	Chile	BHP/Rio Tinto/JECO	(180,000)	2007	World’s largest copper mine; with transition to chloride leach unknown contribution of bioleaching
Lomas Bayas	Chile	Glencore	74,100	1998	Includes production from Antapaccay which is not a bioleach operation
Punta del Cobre	Chile	Sociedad Punta del Cobre (Pucobre)	9,000	1994	Biocobre plant may have oxide leach feed not resulting from bioleach
Radomiro Tomic	Chile	Codelco	10,000		Dump 2 low-grade ROM bioleach
Spence	Chile	BHP	(115,000)	2007	Switched to chloride leach cathode production not attributed to bioleach
Cerro Verde	Peru	Freeport McMoran/SMM/Buenaventura	(100,000)	1996	Known to stimulate acid leach with microbial inoculum bu not strictly a bioleach operation
Dexing Copper	China	Jiangxi Copper	2,000	1997	Dump bioleach of low-grade ore 0.05–0.25% Cu
Zijinshan	China	Zijinshang Copper	20,000	1998	First commercial bioleach in China commissioned in 2006 80% recovery in 200 days
Iranian Babak Copper Co	Iran	IBCCO	50,000	2020	Shar-e Babak deposit in Kerman province Au and Ag also produced
Chambishi	Zambia	Zambia Consol. Copper Mines	10,000	2011	20% increase in recovery attributed to bioleach

It can be argued that adoption of SX-EW across the industry enabled the exploitation of relatively low-grade deposits (< 0.5% Cu by weight) through heap and dump leaching (Kordosky 2002) and by extension, bioleaching. Higher-grade deposits can be processed more efficiently through direct smelting or smelting of flotation concentrates. Neale et al. (2011) provided in-depth techno-economic assessments of copper grades, mineralogy, and ore deposit size to identify where bioleaching of whole ores and concentrates for copper production was best suited. Those assessments still largely hold true today although the recent spike in copper price may improve the profitability of scenarios that were sub-economic in 2011.

The commitment to bioleaching technology in Chile was demonstrated during the period 2002 to 2016, during which a major investment was made through investments in Chilean and Japanese universities and the formation of the biotechnology company BioSigma S.A. This entity was formed through an alliance between Codelco and Nippon Mining and Metals Company Ltd. with the intent of focusing advanced biotechnological tools including genomics, proteomics, and bioinformatics to develop and commercialize new strategies for bioleaching of low-grade copper sulfide ores. By 2014, BioSigma claimed 82 biomining-related US and foreign patents. These patents covered novel iron and sulfur-oxidizing microbial isolates, cultivation methods, oligonucleotide-based detection methods, and use of microbial products to promote bioleaching. The scale-up and deployment of a chalcopyrite bioleaching strategy at Radomiro Tomic was estimated to increase recovery of copper by up to 30% compared to conventional leaching. Full-scale deployment of this approach for low-grade run-of-mine (ROM) ore in Dump 2 at the mine received a positive environmental assessment from the Antofagasta regional commission in 2021 and the US $882 million project is expected to begin contributing to extended life of mine by 2023. The SX-EW plant at Radomiro Tomic has a current copper cathode capacity of 10,000 t/year. Nevertheless, in 2016, JX Nippon Mining and Metals transferred its shares of BioSigma to Codelco, stating that they did not see opportunity to apply the technology within their properties.

An interesting technology development program involving the Universidad Católica del Norte in Antofagasta and funding from the Chilean government and the Escondida mine (joint venture between BHP Billiton, Rio Tinto and JECO Corporation, a Japanese consortium) has been underway for over 10 years to develop a monitoring and control system for bioheap leaching (Demergasso et al. 2010). Escondida is the largest copper mine in the world by production (1.19 million tonnes in 2020). The expert control system incorporates biomarkers for key microbial species including *Acidthiobacillus* (*At.*) species, *Leptospirillum ferriphilum* and *Sulfobacillus* species CBAR13 (Marín et al. 2021). These biomarkers were validated to provide control indications for aeration, pH control, and nitrogen to improve bioleaching performance of the Escondida operations.

The top five copper-producing nations can point to successful heap leach operations that have accounted for much of their success. However, as the copper mineralogy has changed with depth to increasing sulfide content and hypogene deposits, the efficacy of acid (or bio) leaching alone has declined and flotation concentration has emerged as the process of choice (Ghorbani et al. 2015). In fact, new project planning by Cochilco included at least three bioleach projects between 2010 and 2015 (Cochilco 2010), but not any bioleach project in the period 2018–2027 (Cochilco 2018). The increasing trend for reliance on copper concentrate production (73.3% of new projects) compared to SX-EW and heap leaching (0.7%) is also reflected in these publications. Provided the concentrates are relatively free of deleterious elements, the discount between concentrate and cathode prices is negligible once operating and capital costs are factored in. Moreover, flotation returns a saleable product rapidly without the need for extensive area within a mine on which to operate heap and dump leaching. These leach areas are also frequently built upon double-liner geotextile membranes to collect the acidic, metal-laden leach solutions and prevent escape to the environment, adding to the cost of these operations. It is important to remember that beyond the need to have suitable mineralogy to support bioleaching, the longer time required for bioleaching has also been a substantial barrier to deployment of this technology (Johnson 2018; Sheffel 2006).

The change in copper mineralogy towards refractory primary mineral sulfides such as chalcopyrite with increasing depth in porphyry copper ore deposits coupled with water scarcity (and local community concerns to assure water quality) has also driven a transition to the use of brackish water or even sea water to leach copper, as the chloride required for efficient leaching may exceed the concentration of that in sea water by a factor of 3 or more. Copper leaching with chloride has several positive advantages when compared to sulfate-based acidic systems (including bioleaching), including higher dissolution rates, reduced passivation of the mineral surface and improved economics derived from the ability to use sea water or even desalination brine, and greater acceptance by regulatory authorities concerned about depletion of freshwater sources (Toro et al. 2021). It has been projected that sea water will account for nearly 50% of the water used in mining in Chile by 2030 (Cochilco 2020). This is a significant change that has an existential impact on bioleaching as a processing alternative, since it has been widely demonstrated that chloride has a toxic effect on many classes of acidophilic bioleaching microorganisms. While SX-EW operations can be adapted to chloride-containing leach solution, this transition has led to a dramatic decline in copper cathode production attributable to bioleaching. For example, at Carmen de Andacollo in Chile, copper cathode production has declined from 58,000 tonnes/year to only 1000 t/year as the switch has been made to primarily concentrate production. This 98% reduction can likely be extended across the industry. As a consequence, the tables of commercial copper heap bioleaching operations that appeared in the 2013 minireview (Brierley and Brierley 2013) and other reviews (Brierley 2008; Neale et al 2011; Schippers et al. 2014) have been condensed in Table 1 here to reflect the reduced production of copper from bioleaching around the world.

The de-emphasis on development of new biotechnology innovations in Chile is symptomatic of the apparent decline in use of bioleaching in the copper industry. However, it should be noted that coincident with the reduced utilization of heap bioleaching for low-grade copper extraction, the use of tank bioleaching with moderately thermophilic bacteria has been optimized at Iran Babak Copper Company (IBBCo) to produce 50,000 tonnes of cathode copper per year. Historically, Alliance Copper (a joint venture of Codelco and BHP Billiton at the time) demonstrated tank bioleaching of chalcopyrite concentrate at elevated temperature (design operating temperature of 78 °C) in the BioCOP^®^ process at the Chuquicamata mine using thermophilic archaea. This prototype plant comprised six 1260 m^3^ reactor vessels and was commissioned on August 2003 and operated through 2005 to produce 20,000 tonnes of copper cathode per year. Unfavorable economics ultimately halted further development and commercial implementation of a full-scale plant to process 490,000 tonnes of concentrate per year. Presumably, the unique economics of bioleaching concentrate in Iran compared to smelting have allowed that process to go forward.

**Fig. 1:**
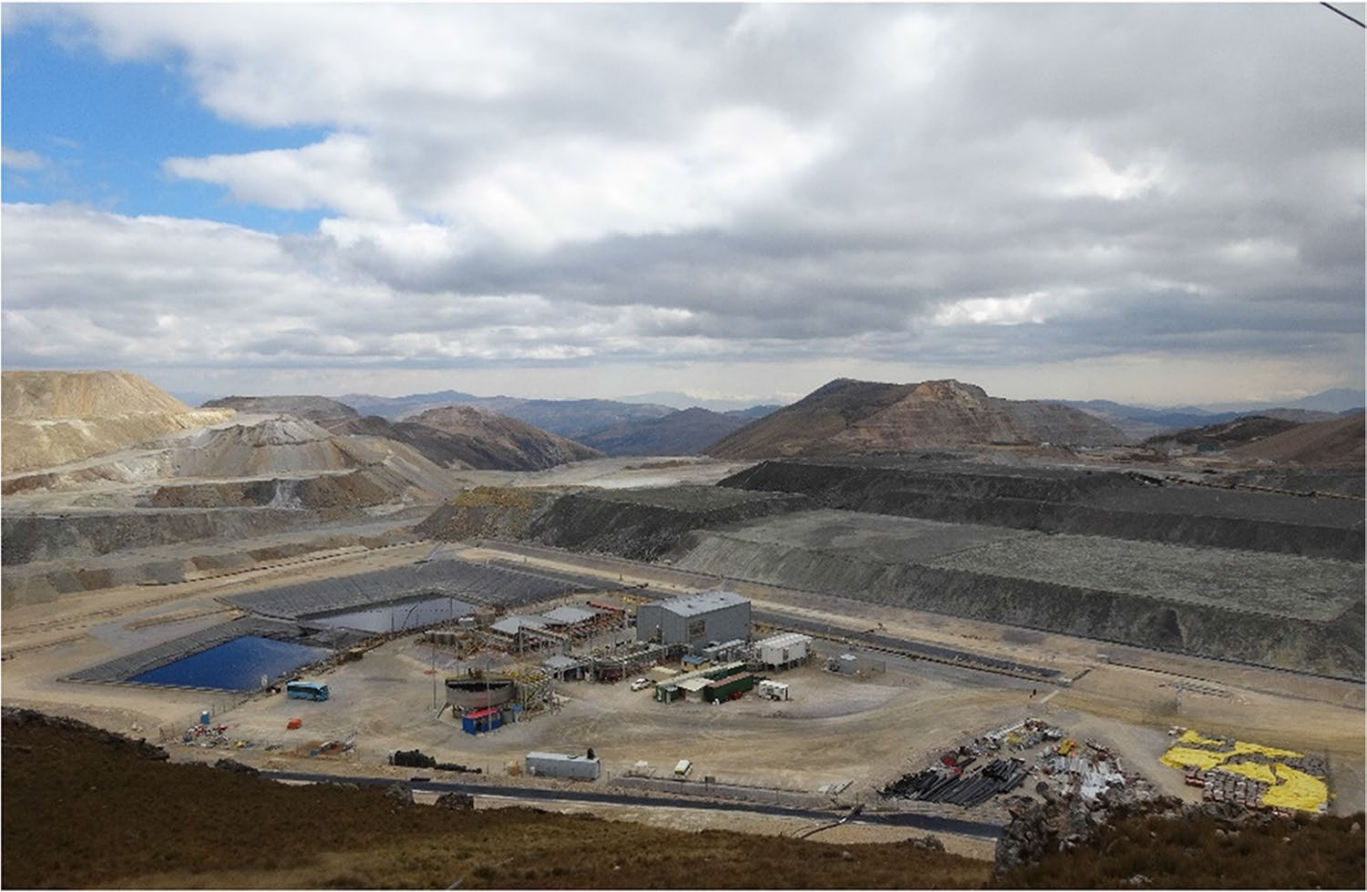
Overview of the Yanacocha enargite bioleach demonstration during operation, including solution ponds, SX-EW plant and leach pads (left to right)

Newmont’s Yanacocha mine demonstrated commercial-scale bioleaching of enargite (Cu_3_AsS_4_)-dominant copper ores also containing chalcocite (Cu_2_S) and covellite (CuS). The 1 million tonnes demonstration (depicted in Fig. 1) examined heap (crushed ore, conveyor stack and aeration) and dump (ROM, truck dumped, no aeration) bioleaching using a mixed consortium of mesophilic and moderately thermophilic bacteria and thermophilic archaea between 2013 and 2017. A total of 2670 tonnes (5.9 million lb) of copper cathode was produced in a small SX-EW plant onsite by the conclusion of the project. Over the course of the operation, planktonic bacterial populations underwent a transition from *Acidithiobacillus* to *Leptospirillum* (Roberto 2018). Internal heap temperatures above 50 °C were observed through much of the operation, but while moderately thermophilic bacteria were not observed in solution (*Sulfobacillus* and *Ferrimicrobium*, for example), they were found in abundance among sequences retrieved from solid residue samples. Archaeal sequence analyses revealed the emergence of apparent native strains of *Ferroplasma* that dominated the archaeal population by the end of the demonstration. *Ferroplasma* has been observed in other copper leach operations (Ruan et al. 2013) where elevated temperature (45–60 °C leach solution, > 60 °C internal heap temperature), high ferric iron (> 30 g/L), and sulfuric acid (> 10 g/L) have exceeded those concentrations seen in other commercial heap bioleach operations. It is anticipated that this process will be deployed at much larger scale as the mine extends life to exploit increasing amounts of sulfides including enargite.

A recent comprehensive review of bioleaching in China (Yin et al. 2018) included 21 different mine sites where bioleaching had been evaluated, but only reported on production levels for the Dexing and Zijin mines, with an aggregate production of approximately 20,000 tonnes of cathode per year. Like the techno-economic assessment presented by Neale et al. (2011), these authors concluded that the relatively low-grade ore found in China (average 0.87% Cu nationally) in predominantly small-scale mines (88%) was well-suited to bioleaching. The Zijin combined gold and copper operation was recently reviewed (Chen et al. 2020) and reported effective bioleaching of low-grade ore with an average grade of 0.43% Cu and a cutoff grade of 0.15%. The Zijin mine was the first commercial copper bioleach operation in China. Similar to the Yanacocha enargite bioleach demonstration in Peru, the Zijin operation experiences extreme solution chemistry, with a pH ranging from 0.8 to 1.0, elevated temperature between 45 and 60° year-round, and total dissolved iron > 50 g/L. These conditions were noted to lead to the inhibition of mesophilic iron-oxidizing bacteria such as *Acidithiobacillus* and *Leptospirillum*, and the dominance of moderately thermophilic sulfur-oxidizing bacteria such as *Acidithiobacillus caldus* and *Acidithiobacillus albertensis* (Xingyu et al. 2016). The authors remark that efficient bioleaching progresses despite a low planktonic cell count of 10^4^ cells/mL while still achieving nearly 80% copper recovery by SX-EW. And similar to the transition in Chile, about 80% of the copper recovery at the Zijin mine is now produced as a high-grade flotation concentrate (~ 22% Cu), with ore containing > 0.25% Cu diverted to the flotation circuit and the lower-grade material placed on the bioleach heaps. The ore mineralogy is reported to contain 5.8% pyrite, 0.2% digenite (Cu_9_S_5_), 0.2% covellite, 0.1% enargite, and 0.03% chalcocite by weight which explains some of the observed operating parameters and the challenges for a strictly bioleach-based extraction process. The Chinese government also described (Yin et al. 2018) to have supported several major research programs to develop bioleaching technologies in China, with a new major initiative supporting in situ bioleaching of copper, gold, and uranium, e-waste recovery of copper, use of renewable energy, and environmental remediation.

While lacking defined studies of the microbiology associated with their commercial leaching operations, it should be noted that major copper mines including Quebrada Blanca, Cerro Verde, Bingham Canyon, and Morenci in Chile, Peru, and USA, respectively, have all reported the benefits of improving aeration and optimizing heap irrigation while monitoring chemical species indicative of microbial activity and iron and sulfur oxidation. As these reports are now more than 10 years old, the transition to concentrate production at these mines is certain and the contribution of bioleaching to production impossible to estimate from current information.

The only new project proposed recently to employ bioleaching for copper is the Haib mine in Namibia, owned by Deep-South Resources. The copper porphyry deposit is estimated to contain between 2 and 3 billion lb (909,000–1.4 M tonnes). However, the mineralogy is dominated by chalcopyrite which requires high-temperature leaching to achieve reasonable copper recovery. Advancement of the project was put into question when the Namibian Minster of Mines and Energy refused to renew the exclusive prospecting license in 2021. The status of the license remains under litigation in Namibia in 2022.

## Bioleaching of cobalt, nickel, and zinc

Only the Terrafame-mixed metal sulfide plant in Finland is producing economic quantities of cobalt using heap bioleaching after the Kasese Cobalt Company tank bioleach ceased operation at the Kilembe copper mine upon exhaustion of the available stockpile of Co-rich pyritic tailings in 2013. The latter process at its peak produced about 1100 tonnes per year of cobalt cathode. That quantity represented approximately 0.8% of global production in 2020. The Terrafame Sotkamo (Talvivaara in 2013) mine in Finland is estimated to be producing approximately 40% of a maximum 1500 tonnes per year (600 tonnes) that would represent 0.4% of global Co production in 2020. Terrafame reported production of 29,600 tonnes of nickel, representing 1.2% of global production (Tuomela et al. 2021) and 55,100 tonnes of zinc representing 0.4% of global production.

Also in Finland, Mintek and the former Mondo Minerals (now Elementis Minerals) designed, built, and operated a nickel concentrate tank bioleach project (Neale et al. 2017) for 3 years using moderately thermophilic bacteria on a complex polymetallic feed from the Vuonos and Sotkamo talc concentrators. Seven 112 m^3^ stirred-tank bioreactors bioleached 35 tonnes per day of concentrate ground to a P80 = 20 µm with a 7-day residence time and 15% solids content. The commercial operation was planned to produce 1000 tonnes per year of nickel and 20 tonnes of cobalt but was placed in care and maintenance with the change in ownership in 2018 for economic reasons. The European Commission through the Horizon 2020 program has continued to look at the potential for such projects to contribute to goals of a circular economy for metals (Mäkinen et al. 2020, 2021). Recent work has examined both primary concentrates and concentrates produced from tailings. Table 2 summarizes current and projected production of nickel, cobalt, and zinc from bioleaching in Finland.

**Table 2:** The 2020 (Terrafame) and prospective (Elementis) production from bioleaching. Elementis production estimated by Finnish government (Tuomela et al. 2021)

Mine	Location	Operator	Metal production, t/y	Year Initiated	Notes
Terrafame	Sotkamo Finland	Finnish Minerals Group, Galena, Sampo	28,740 Ni 55,100 Zn 600 Co	2011	Metals precipitated as sulfides; integration of bioenergy plant further reduces carbon emissions footprint; plan for uranium production in the future
Vuono/Sotkamo talc mines	Finland	Elementis	(1000 Ni 20 Co)	2015	Mondo Minerals predecessor to Elementis operated 35 t/d bioleach plant at Vuonos concentrator for 3 years now in care and maintenance

## Gold recovery via biooxidation

Gold production from biooxidation of sulfidic ores and concentrates has emerged as the clear and enduring success story for industrial application of this biotechnology as this mini-review was being prepared. Bioleaching of base metals differs from biooxidation of gold in an important way. While base metals may be dissolved in solution through biological action of iron and sulfur-oxidizing microorganisms (rendering those metals amenable to recovery through ancillary processes like SX-EW), gold remains insoluble until a suitable lixiviant (typically cyanide) is applied to biooxidized ore or concentrate residues. The role of the microbes in the case of gold is to liberate gold from sulfidic mineral matrices like arsenopyrite to increase the access of the gold lixiviant. Additional separation and neutralization steps are often required to provide the right conditions for dissolution of gold, after which recovery of gold-cyanide or other complexes (thiosulfate has been applied commercially and may also be applied to acidic solid residues with little or no neutralization required) can be achieved with activated carbon or resins.

Perhaps the most significant development since 2013 which demonstrates the maturity and commercial acceptance of gold biooxidation for refractory gold-bearing concentrates was the acquisition of the Biomin BIOX^®^ process by Outotec (now Metso Outotec) in 2015. This industrial giant serving the mining industry has financial resources, technical expertise, and commercial products across the mineral processing spectrum that are being leveraged to improve both the application and efficiency of the BIOX^®^ oxidative pre-treatment technology. Along with BIOX^®^, Metso Outotec also provides the proprietary ASTER process for detoxification and removal of cyanide degradation products like thiocyanate, the HiTeCC high-temperature process for mitigation of gold losses due to gold-robbing when treating high organic carbon ores or concentrates, and MesoTHERM^®^, a high-temperature microbial process utilizing thermophilic microorganisms to reduce cyanide consumption and cooling requirements for refractory gold ore biooxidation. Except for the HiTeCC process, which involves physical stripping of Au-CN complexes from carbon to exchange to fresh-activated carbon, the ASTER™ and MesoTHERM^®^ processes are microbiological. A recent presentation by Jan van Niekerk (Haileybury School of Mines, Dec. 8, 2021) suggests that with the maturity of the BIOX^®^ product line, they are now turning their attention to base metal concentrate leaching.

ASTER™ (activated sludge tailings effluent remediation) was developed to reduce or eliminate residual cyanide and cyanide byproducts in process solutions coming from the BIOX plant. These effluents include oxidation products formed by reaction of polysulfide and thiosulfate produced during biooxidation of sulfide concentrates and cyanide, including thiocyanate. Thiocyanate has been demonstrated to have toxic effects on bioleaching microorganisms at concentrations as low as 1 ppm, and levels of thiocyanate exceeding 5000 ppm have been observed in some BIOX^®^ circuits (van Niekerk and van Buuren 2020). In order to promote the ability to recycle process water and ensure environmental discharge limits can be achieved, an activated sludge-based bioprocess was developed (van Buuren et al. 2011) with the first commercial installation at the Consort mine in South Africa starting operation in 2010. This plant treated an influent concentration of 120 mg/L of thiocyanate and 10–30 mg/L cyanide at a rate of 320 m^3^/day in 20 m^3^ bioreactors. With the success of this plant, a larger plant treating 800 m^3^/day in 200 m^3^ bioreactors with thiocyanate design feed concentration of 3500 mg/L was commissioned at the Suzdal BIOX^®^ plant in Kazakhstan. Typical effluent thiocyanate and cyanide concentrations are less than 0.1 mg/L. The ASTER™ circuit at the Fosterville mine in Australia was commissioned in 2020 and is treating 3000 m^3^/day at the highest incoming thiocyanate concentration among operating BIOX^®^ plants at 5000 mg/L in 400 m^3^ bioreactors (van Niekerk and van Buuren 2020).

The most recent technical development within the BIOX product line is the MesoTHERM^®^ biooxidation process. High cyanide consumption has been a techno-economic challenge for the BIOX^®^ process in the past, due to residual reactive sulfur species which consume cyanide and produce thiocyanate. Batch test work revealed that a combined mesophile-thermophile sequenced treatment of gold-bearing sulfide concentrate could reduce cyanide consumption after biooxidation by nearly 50% through more efficient biooxidation of reduced sulfur species to sulfate and greatly reduced formation of thiocyanate during cyanidation of the biooxidized concentrate residue. In contrast to the mesophilic consortium (comprised of *Leptospirillum ferriphilum*, *At. caldus*, *Thermoplasma* spp., *Ferroplasma acidiphilum*, and *Acidiplasma* (*Ap*.) *cupricumulans*), the thermophile consortium was initially dominated by *Ap. cupricumulans* and then *Metallosphaera* spp. as temperature increased to 70 °C (van Niekerk and van Buuren 2020). In practice, the MesoTHERM^®^ process incorporates a second stage of biooxidation taking the mesophilic bioreactor slurry at 40 °C and increasing the temperature of the thermophilic bioreactors to 65 °C. Scale up of this combined process has demonstrated sustained reduction in cyanide consumption in the gold leaching circuit downstream after neutralization and thickening. A commercial-scale MesoTHERM circuit has been running at the Fairview plant in South Africa since 2019.

Since the central mesophilic BIOX^®^ process has been reviewed in many publications, including this mini-review series, and on a regular basis (e.g., van Niekerk and van Buuren 2020) by the technology developers and the current owners of the process, it will not be described here except to say that total cumulative gold production attributed to BIOX^®^ is now in excess of 30 million ounces (933 tonnes) over 30 years of commercial deployment. Approximately 1.1 million ounces was produced by BIOX^®^ plants in 2020, equivalent to 0.9% of global gold production that year.

Although it is widely recognized that China is the largest global producer of gold, there is limited transparency regarding the extent to which biooxidation of refractory gold concentrates contributes an unknown proportion to an estimated 11 million ounces (10.6% of 2020 global production). Besides the Jinfeng mine which utilizes BIOX^®^ technology, five other mines in China — Axi, Jinchiling, Laizhou, Sanhe, Tianli and Yantai Gold — have been identified that have evaluated or use biooxidation technology to treat refractory gold concentrates. These mines account for about 1000 tpd of aggregate concentrate biooxidation capacity, compared to Jinfeng’s throughput of 790 tpd.

The Polyus Olimpiada mine in Krasnoyarsk, Siberia, Russia, has operated a biooxidation plant treating refractory gold flotation concentrates since 2001 (Belyi et al. 2018). Unlike many biooxidation plants using the BIOX^®^ process, the Olimpiada mine operates a proprietary biooxidation process known as BioNORD^®^. The first plant treated a 16% concentrate slurry round to a P100 < 40 µm at a rate of 450 t/day. This process has undergone continuous refinement including larger bioreactor tanks (1000 m^3^), expanded lines of bioreactors, process automation, and more efficient impellers to increase aeration and maintain suspension of the concentrate slurry. Because of the colder ambient temperatures experienced at the plant, it was possible to reduce active cooling of the process by placing much of the bioreactor tankage outside. The combined improvements increased the operating slurry density to 22%, improved sulfide oxidation and gold recovery, daily throughput to 1510 t/d, and reduced energy consumption by at least 25% at an operating temperature of 38–40 °C. The bioreactor consortium is dominated by *Acidiferrobacter* spp. and *Ferroplasma* spp. (over 50%) with *Acidithiobacillus* and *Acidiphilium* spp. making up another 26% of the population. Polyus reported production of over 30 tonnes of gold through the BIONORD process at Olimpiada in 2019, representing about 0.83% of global gold production that year.

As previously reported in the 2013 mini-review (Brierley and Brierley), Newmont operated a commercial whole-ore biooxidation process at the Carlin mine in Nevada (described as the Refractory Leach Project at Newmont). The process operated for 10 years (Tempel 2003; Roberto 2017) treating 800,000 tonnes per year of refractory gold ore and producing an estimated 500,000 oz of gold over 10 years. The patented Newmont process trademarked as BioPro™ was applied to refractory ore in an “on/off” leach pad cycle that required removal of the biooxidized ore prior to neutralization and subsequent cyanidation to recovery gold. Interestingly, this operation also experienced extremes of pH (1.3), temperature (81 °C), and dissolved iron (59 g/L) although these were not anticipated in laboratory and pilot scale work.

Table 3 summarizes global gold production from the various proprietary and unknown biooxidation processes operating around the world in 2020 accounting for over 66 tonnes of gold.

**Table 3:** Summary of gold production from biooxidation of refractory gold concentrates

Operation	Location	Owner	Concentrate t/d	Au production, ounces	Year commissioned	Notes
BIOX^®^						
Obuasi	Obuasi, Ghana	Anglo Gold Ashanti	250	200,000	1994	Back in operation after time in care and maintenance
Barberton/Fairview	South Africa	PanAfrican Resources	47	65,000	1986	Birthplace of BIOX^®^ process; MesoTHERM 2019
Fosterville	Victoria, AUS	Kirkland Lake Gold	211	150,000	2005	HiTeCC developed here; ASTER™
Jinfeng	Guizhou, China	China National Gold Group Corporation	790	70,000	2007	First BIOX^®^ plant in China
Kokpatas	Uzbekistan	Navoi Mining and Metallurgical Combinat	2138	432,000	2009	Production estimate; operates from -20 °C to 50 °C
Runruno	Philippines	FCF Minerals Corporation	404	90,000	2016	ASTER™ and HiTeCC
Suzdal	Kazakhstan	Nordgold	520	90,000	2005	Operates from − 40 to 45 °C; ASTER™and HiTeCC
Cam and Motor	Zimbabwe	RioZim	100/200	(75,000)	2022	Phase 1 commissioned (annual production estimate); ASTER™
BIONORD^®^						
Olimpiada	Krasnoyarsk, Russia	Polyus Gold	1500	965,000	2001	
Unknown						
Axi	Xinjiang, China	Yining (Ghulja) Co	130		2004	
Jinchiling	Zhaoyuan, China	ZhaojinMining Industry Co	100		2007	
Laizhou (BioGold)	Shandong, China	Sino Gold Mining	200	75,000	2001	Only reported production information available
Sanhe	Jiangxi, China	Jinshan Gold/Huaqiao Gold	100		2006	
Tianli	Liaoning, China	Liaoning Tianli Gold	250		2003	
Yantai Gold	Shandong, China	Yantai Gold	130		2000	

## Global contribution of bioleaching and biooxidation to non-ferrous metals production

The impacts of the COVID-19 pandemic were taken into account in whether to use the most recent production data available (2020) or utilize 2019 information since comprehensive 2021 data was not yet available. The pandemic did not result in a decrease in production for cobalt and zinc, for which increases of 1.4% and 1.5% occurred, while nickel, copper, and gold production declined by 7.4%, 2%, and 3.3% respectively, year over year.

For copper, the “typical” SX-EW plant producing copper cathode from bioleaching has been on the order of 10,000 t/year, with some multiples of this scale observed. The recently commissioned IBBCo copper concentrate bioleach operation in Iran appears to be the largest plant in operation at this time, producing 50,000 t/year of copper cathode. Due to the previously described process changes in Chile, the Radomiro Tomic mine may account for the largest amount of copper attributable to bioleaching, with all other operations having declined substantially since 2010 (Table 4).

**Table 4:** Global production of non-ferrous metals via bioleaching and biooxidation (Bio) compared to total global production (total) for the years 2019 and 2020, and share of Bio production

	Production, tonnes*			
Metal	Total 2019	Total 2020	Bio	Bio share %
Nickel	2.7 million	2.5 million	29,600	1.2
Cobalt	140,000	142,000	600	0.4
Copper	20.4 million	20 million	232,719	1.2
Zinc	13.5 million	13.7 million	55,100	0.4
Gold	3597.2	3478.1	66.6	1.9

^*^Global production numbers from statista.com except for gold, from World Gold Council

## Sustainability of bioleaching and biooxidation

Bioleaching technology has long been touted as a green technology owing to its basis in biotechnology, reduced use of chemicals, production of waste materials, and perhaps reduced energy consumption. The latter has been a major focus of engineering efforts focused on improving bioleaching and biooxidation, particularly in the BIOX^®^ and BioNORD^®^ processes, where more efficient impeller designs have had a dramatic effect. The ASTER™ process also provides a means to recycle water effectively in refractory gold concentrate biooxidation.

The Terrafame bioleaching operation in Finland recently concluded an independent, verified lifecycle analysis of its nickel sulfate process compared to the average carbon footprint of nickel production benchmarked by the Nickel Institute. The conclusion was that 1 kg of nickel sulfate produced by Terrafame emitted only 32% of the CO_2_ compared to conventional processing, and that Terrafame’s annual nickel sulfate production prevented emission of 620,000 tonnes of CO_2_ (Terrafame 2020). So while bioleaching has remained a niche technology in the mining industry, the increasing imperative to achieve net zero science-based CO_2_ emissions targets by 2050 by most major mining companies may lead to expansion of this technology in the next decades.

## In situ leaching

In situ leaching or in place leaching has also been termed in situ recovery and recently reviewed by Seredkin et al. (2016) and Kaksonen et al. (2018). Where microorganisms are involved, the term in situ biomining has been introduced (Johnson 2015). In situ leaching has been applied since the late 1950s for uranium recovery, providing today about 50% of the world uranium production from ores. Subsurface microorganisms may play a role in this process by oxidation and/or reduction activities (Zammit et al. 2014; Richter et al. 2018). Later, attempts for in situ recovery of base metals have been undertaken. In the in situ leaching process, a leaching solution is injected into a subsurface ore body where valuable metals such as uranium or copper are dissolved from the ore. The pregnant leach solution (PLS) is pumped to the surface where metal extraction takes place. Afterwards, the solution is re-injected in the underground. A major advantage of in situ leaching is that metal extraction takes place without conventional mining. Crushing and milling of the ore and deposition of waste rock and tailings are avoided, minimizing costs and the environmental footprint. Critical parameters for successful in situ leaching operations are the permeability of the ore body, the hydrogeology and control of the leach solution, and the leachability of the desired metal. The permeability could be increased by blasting or hydraulic fracturing of the ore body.

In situ leaching has a huge potential for low-grade ore deposits and deeply buried ore bodies (> 1 km below the land surface) for which conventional mining and processing is uneconomical. Besides uranium (U), the commodities gold (Au), copper (Cu), nickel (Ni), rare earth elements (REE), and scandium (Sc) have been successfully recovered via in situ leaching (Seredkin et al. 2016).

In situ biomining has been suggested as a remedy for the ore losses originating from block cave mining applied to huge porphyry copper deposits. Because of the increasing dilution with waste rock in the case of an advanced block caving operation, up to 25–30% of the ore remain in situ and represent an economic target for biomining (Schippers et al. 2014).

The importance of microorganisms for in situ leaching processes is under debate. The availability of molecular oxygen needs to be considered. In contrast to heap or tank bioleaching or biooxidation, where sufficient molecular oxygen is supplied for acidophilic ferrous iron and sulfur compound-oxidizing bacteria and archaea via active aeration, the availability of molecular oxygen in subsurface ore bodies is low. The content of molecular oxygen in the injected leaching solution is limited by its low solubility as a function of temperature and pressure (Richter et al. 2018), which increase with depth. If the in situ leaching operation occurs in a smaller fractured ore body in an existing aerated mine, molecular oxygen is available (e.g., Sand et al. 1993). However, in deeply buried ore deposits at greater depth, aerobic acidophiles likely do not play an important role for in situ leaching. Ferric iron in acidic solution is usually the chemical leaching agent, and when injected, becomes an electron acceptor (instead of molecular oxygen) for sulfur-compound oxidizing acidophiles such as *Acidithiobacillus ferrooxidans* under anaerobic conditions. This process has been studied in laboratory experiments at elevated pressure by Zhang et al. (2018).

**Fig. 2:**
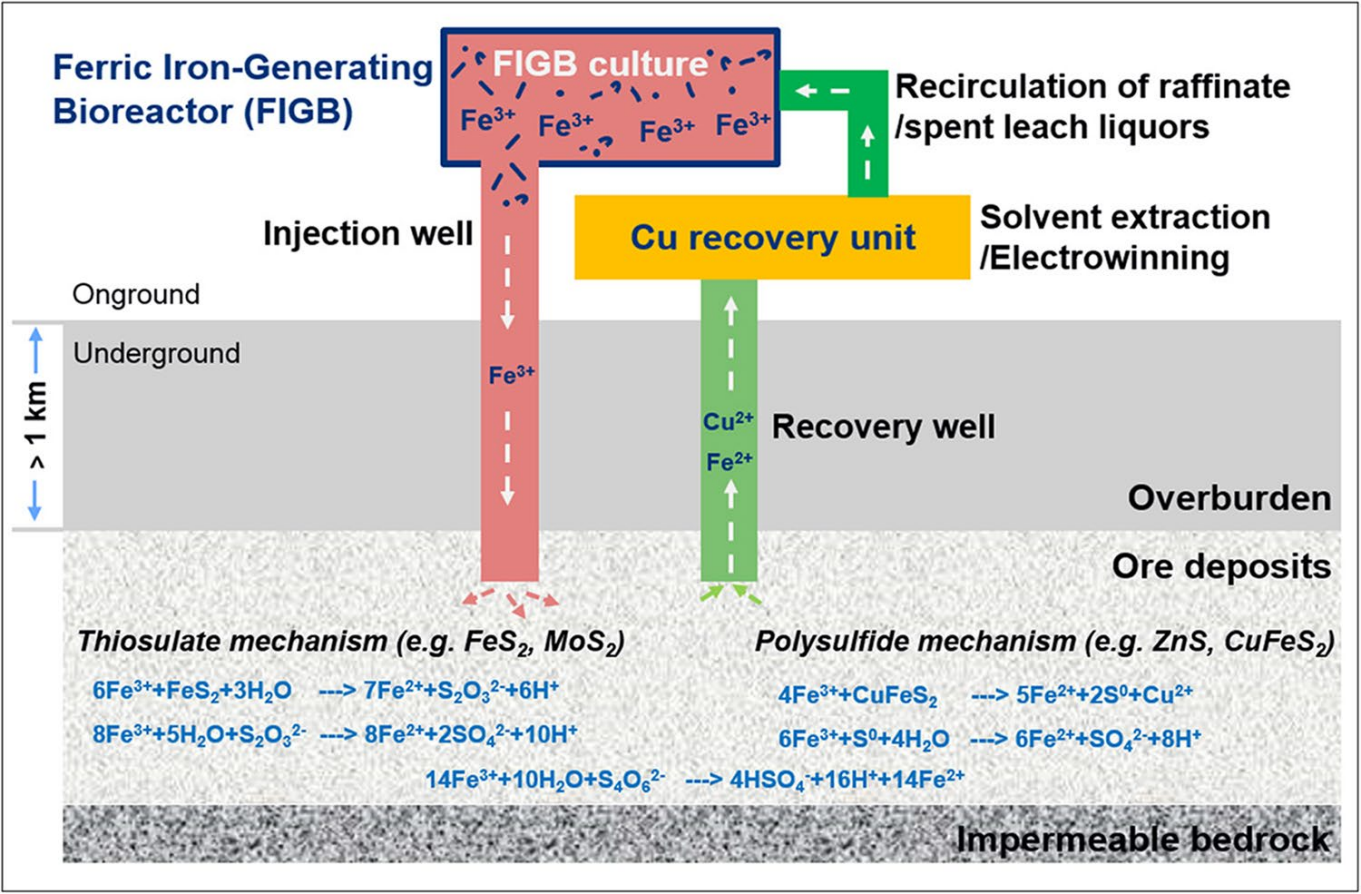
Scheme of in situ leaching for base metal recovery involving ferrous iron oxidizing acidophiles grown in a bioreactor (taken from Zhang et al. 2018, modified from Johnson 2014, with permission from the publisher)

The ferrous iron in the “consumed” leaching solution can either be re-oxidized by addition of chemicals such as hydrogen peroxide (H_2_O_2_; Richter et al. 2018), or by ferrous iron-oxidizing acidophiles in a ferric iron-generation bioreactor (FIGB, Fig. 2). The concept of combing a FIGB with in situ leaching of copper from a fractured ore body has been investigated in the European Horizon 2020 research project BIOMOre at laboratory and pilot scale (Pakostova et al. 2018; https://cordis.europa.eu/project/id/642456).

After termination of an in situ leaching operation, an ongoing dissolution of metals and a potential release of metal-rich leaching solutions need to be minimized and controlled. A partial solution is to diminish microbial activity. Approaches for eliminating bacteria introduced during in situ bioleaching of fractured sulfidic ores in deep subsurface have been tested. A high chloride concentration of 65 g /L (Bomberg et al. 2018) and a “decommissioning solution” containing chloride, formate, and some other compounds (Ballerstedt et al. 2017) have shown to be successful for inactivating bacteria for an effective termination of in situ biomining.

## Acid and reductive bioleaching of laterite ores

While oxidative bioleaching of sulfide ores is an industrial reality (biomining), biotechnological processing of silicate and oxide ores only exists at laboratory scale. Most advanced is the bioleaching of laterite ores for the recovery of mainly cobalt (Co) and nickel (Ni) recently reviewed by Marrero et al. (2020) and Santos and Schippers (2022). Laterite ore deposits constitute 60 to 70% of the world’s Ni resources and are complex in their structure mineralogy. They originate from weathering processes in tropical climate and comprise a lower zone of silicate-rich saprolitic laterite (often rich in magnesium silicates), and an upper oxidized zone of limonitic laterite, consisting of iron oxides dominated by goethite (α-FeOOH) or limonite (FeOOH·nH_2_O) and manganese oxides such as asbolane (Mn^4+^ (O,OH)_2+_·(Co,Ni,Mg,Ca)_x_(OH)_2x_·nH_2_O) and lithiophorite (Al,Li)(Mn^4+^,Mn^3+^)O_2_(OH)_2_) (Butt and Cluzel 2013). Laterite ores contain valuable metals including Ni and significant proportions of Co, Sc, V, and Cu. The Ni extraction from the laterite ores is mainly performed using pyrometallurgical techniques, which are able to process only saprolitic laterite ores whereas the processing of limonitic laterites requires hydrometallurgical methods such as HPAL (high pressure acid leaching) or AL (acid leaching). The latter however require high energy or reagent consumption and expensive capital equipment costs and are faced with technical and environmental challenges. Therefore, in most existing mines, limonitic laterite ores are being stockpiled as mining residues. Although it is widely admitted that processing limonite is the solution to meet future demand in Ni and contributing to the supply of Co, Cu, Sc, and V, there is still a lack of novel and sustainable robust processing routes allowing reduced energy and reagent inputs and producing harmless residues. Here, biohydrometallurgy may come into play.

Several studies employed bioleaching of both saprolitic as well as limonitic laterite ores, as well as laterite tailings by organic acids generated by heterotrophic bacteria or fungi (Bosecker 1977, 1986; Coto et al. 2008; Chaerun et al. 2017; Giese et al. 2019; Nasab et al. 2020). However, these microorganisms require the addition of organic carbon probably as processed waste from the agricultural or food industries. This makes control of bioleaching with organoheterotrophs more costly than bioleaching with lithoautotrophic acidophiles, and undesirable microorganisms may disturb these processes under real industrial operation conditions. Also bioleaching with sulfuric acid generated by the oxidation of added elemental sulfur by *At. thiooxidans* was successfully tested (Coto et al. 2008; Jang and Valix 2017).

While bioleaching with inorganic or organic acid-producing microorganisms seems to be the only bioprocessing route for saprolitic laterites, reductive bioleaching offers a promising route for bioprocessing of limonitic laterite ores. In these ores, the valuable metals Ni and Co are bound to several mineral phases including iron(III) or manganese(IV) oxides, and especially the latter can be dissolved via reductive bioleaching. Reductive bioleaching means dissolution of an ore or another solid material by a chemical reduction reaction catalyzed by microorganisms. Reductive bioleaching of limonitic laterites using *At. ferrooxidans* has been demonstrated at laboratory scale and described as the Ferredox process (du Plessis et al. 2011; Hallberg et al. 2011; Ñancucheo et al. 2014; Johnson and du Plessis 2015; Smith et al. 2017; Santos et al. 2020). Here, a main reaction is the dissimilatory reduction of ferric iron coupled to oxidation of added elemental sulfur, which can be catalyzed by several acidophilic bacteria besides *At. ferrooxidans* (Brock and Gustafson 1976; Schippers et al. 2014; Johnson et al. 2017). The Ferredox process has been proposed to treat limonitic laterite ores for cobalt and nickel recovery by means of anaerobic reductive dissolution with autotrophic acidophilic bacteria. As a modification, aerobic reductive dissolution of laterites with *Acidithiobacillus* species has been demonstrated at low pH < 1, including the use of *At. thiooxidans* as the only organism (Marrero et al. 2015, 2017; Stanković et al. 2022). Since dissimilatory reduction of ferric iron has not been described for *At. thiooxidans* the question about the mechanism of iron oxide reduction arose. One current hypothesis is that intermediary sulfur compounds such as hydrogen sulfide or thiosulfate formed during enzymatic oxidation of elemental sulfur to sulfuric acid, or biologically activated sulfur itself (Marrero et al. 2020; Johnson et al. 2021), serve as chemical reductant for iron and manganese oxides. This was also discussed for enhanced dissolution of seafloor manganese nodules in aerobic bioleaching experiments with *At. thiooxidans* (Kumari and Natarajan 2001). Aerobic reductive bioleaching has advantages over anaerobic reductive dissolution of laterites comprising no requirement for gassing with nitrogen gas to keep an anoxic atmosphere and less acid consumption due to enhanced sulfur oxidation.

However, reductive bioleaching of laterites is possible under anaerobic as well as aerobic conditions at low pH offering two promising bioprocessing options for limonitic laterites (Marrero et al. 2020). The reductive bioleaching technology has the potential to increase metal recovery in existing mines and transform unexploited ores, limonite stockpiles, and even tailings from laterite ore processing into valuable resources. The major hurdles facing reductive bioleaching are on the one hand the low reactivity of iron oxides such as goethite being an important Ni bearing and the main mineral phase in limonitic laterites (Stanković et al. 2022), and on the other hand, the high processing costs for tank leaching operations. Thus, reductive bioleaching in ponds or heap bioleaching should be developed.

In addition to limonitic laterites, reductive bioleaching might be applied as a pretreatment step for processing of refractory gold or platinum group element (PGE) oxide ores (Kaksonen et al. 2014; Hedrich et al. 2020).

Furthermore, an application of reductive bioleaching to extract metals from mining and industrial waste should be considered (Glombitza and Reichel 2014). Al and rare earths elements have been extracted from red mud by a two-step aerobic and anaerobic bioleaching approach. *Acidianus manzaensis* anaerobically dissolved jarosites via Fe(III) reduction coupled to sulfur oxidation (Zhang et al. 2020a, b). Also demonstrated has been a reductive dissolution of jarosite by the heterotrophic bacterium *Acidiphilium cryptum* (e.g., González et al. 2020). Cu extraction from mine tailings was achieved by a combination of oxidative and reductive bioleaching (Falagán et al. 2017) as an approach for tailings reprocessing.

## Bioleaching of mining residues (tailings) and industrial waste, and microbial metal recovery from acid mine drainage

Biohydrometallurgy offers approaches for metal recovery from different kinds of residues and wastes from mining and industrial processes, which is termed secondary mining or urban mining.

Reprocessing of mine tailings has the advantage of minimizing the environmental impact of the waste by producing a more harmless residue by extracting remaining valuable metals often not of economic interest at the time of tailings’ deposition. Bioleaching of tailings has been examined at laboratory scale in several case studies. A high extraction of Cu, Co, and Ni from sulfide tailings (Ahmadi et al. 2015; Stanković et al. 2015; Falagán et al. 2017; Altinkaya et al. 2018; Mäkinen et al. 2020; Zhang et al. 2020a, b; Lorenzo-Tallafigo et al. 2022; Zhang and Schippers 2022) and laterite tailings (Marrero et al. 2015; Nasab et al. 2020) could be demonstrated. Also, trace metals such as indium have been targeted (Martin et al. 2015). Economic considerations for reprocessing of mine tailings are rare but are a prerequisite for application (Araya et al. 2021; Drobe et al. 2021).

A commercial application of stirred-tank reactor bioleaching for Co recovery has even been applied to a cobalt-containing pyrite concentrate stockpiled in the former Kilembe copper mine in Kasese, Uganda (Morin and d'Hugues 2007) as mentioned above.

Industrial, metal-rich residues including fly ash from waste or coal combustion, slag, sludge, spent catalysts, and electronic scrap such as printed circuit boards (PCB), and spent batteries have often been processed at laboratory or pilot scale via bioleaching either with ferric iron and/or sulfuric acid producing lithoautotrophic acidophiles such as *Acidithiobacillus* (often with sulfur addition) and/or organic acids (or cyanide) excreting heterotrophic bacteria or fungi (Brombacher et al. 1997; Hoque and Philip 2011; Lee and Pandey 2012; Glombitza and Reichel 2014; Hennebel et al. 2015; Zhuang et al. 2015; Kaksonen et al. 2018, 2020; Abhilash and Akcil 2019; Srichandan et al. 2019; Faramarzi et al. 2020). However, this topic was already raised on the first International Biohydrometallurgy Symposium (IBS) 45 years ago (Ebner 1977). Especially, biohydrometallurgy for PCB processing has been development in laboratory scale bioreactor experiments over the last years, and, e.g., up to over 90% of copper recovery has been achieved (Hubau et al. 2020; Monneron-Enaud et al. 2020; Abhilash et al. 2021; Iglesias-González et al. 2022). Metal recycling based on biotechnology will raise increasing interest in future including economic and environmental process considerations such as energy consumption and carbon footprint. Such data are not yet available.

Biotechnology offers different options for the extraction of metals from metal-rich process waters (pregnant leach solution, PLS), acid mine drainage, and industrial wastewaters. These include biosorption, bioaccumulation, bioprecipitation, biomineralization, and also bioelectrochemistry.

Biosorption describes the sorption of metals on biomass (biological cell surfaces) or biomolecules. If living cells also take up metals into the interior of the cell, this is termed bioaccumulation. Biosorption has been tested for numerous metals, there are numerous publications and patents from mainly laboratory and a few pilot-scale studies as described in review articles (Tsezos 2001, 2014; Michalak et al. 2013; Fomina and Gadd, 2014; Beni and Esmaeili 2020; de Freitas et al. 2019). However, industrial application of biosorption has not been described.

More promising for application is the microbially influenced chemical precipitation of metals (bioprecipitation), which is based on the low solubility product of metal compounds. On the one hand, this includes the removal of iron from acid mine drainage by means of microbial iron(II) oxidation to iron(III)hydroxides such as schwertmannite as demonstrated in pilot scale (Hedrich and Johnson 2014; Reichel et al. 2017). On the other hand, valuable metals such as copper, nickel, zinc, and cobalt can be separated by chemical precipitation using hydrogen sulfide as metal sulfides, with pure metal fractions being obtained by varying the pH of the polymetallic solutions during the precipitation process. The hydrogen sulfide can also be produced by means of microbial sulfur or sulfate reduction with sulfur or sulfate reducing bacteria (SRB); these may either be neutrophiles or acidophiles (Hedrich and Johnson 2014; Sánchez-Andrea et al. 2016; Hedrich et al. 2018; Kaksonen et al. 2020). Commercial applications exist (ThioTeq, operated by the Dutch company Paques BV, and BioSulfide, operated by the Canadian company BioteQ). Acid mine drainage processing with acidophilic sulfate reducers has the advantage of selectively recover valuable metals as metal sulfide precipitates, removes sulfate, and also increases the pH due to the proton-consuming sulfate-reducing reaction.

The above described bioprecipitation often goes along with biomineralization, which is the biotechnical production of minerals from metals in solution. Schwertmannite and the bioprecipitation of metal sulfides are such examples. In addition, some microorganisms have demonstrated the ability to form nanoparticles, consisting of pure metals or metal compounds. The nanoparticles can be formed in the cells (intracellular) and on the cell surfaces (extracellular). So far, successful experiments on the laboratory scale for the synthesis of Au, Ag, Pt, Pd, Se, Te, Si, Zr, and Ti nanoparticles have been carried out (Deplanche et al. 2008; Narayanan and Sakthivel 2010; Hosseini et al. 2013; Castro et al. 2014; Saitoh et al. 2018).

Bioelectrochemistry has been increasingly used for metal extraction from solutions in the laboratory in recent years. The principle is as follows: In a bioelectric system (electro-bioreactor), organic substances are oxidized by heterotrophic microorganisms adhering to an anode (electroactive biofilm). The electrons released at the cathode are used for the electrochemical reduction of metal cations to pure metals in elemental form, e.g., shown for the separation of Cu, Pb, Cd, and Zn from dilute solutions. The separation into pure metals was based on a graded adjustment of the electrochemical potential (Modin et al. 2012). Other metals obtained in this way include Co, Cr, Hg, Ag, and Se (Modin et al. 2012; Hennebel et al. 2015; Nancharaiah et al. 2015). This method could possibly be used to extract metals from acid mine drainage and industrial process waters.

## Data Availability

Not applicable.
